# Responses to Stress among East Malaysian Students: Psychometric Properties of the Responses to Stress Questionnaire

**DOI:** 10.21315/mjms2023.30.2.12

**Published:** 2023-04-18

**Authors:** Hui Yee Lai, Whye Lian Cheah, Helmy Hazmi, Ai Ling Ang

**Affiliations:** 1 Department of Community Medicine and Public Health, Faculty of Medicine and Health Sciences, University of Malaysia Sarawak, Sarawak, Malaysia; 2 Department of Psychological Medicine, Faculty of Medicine and Health Sciences, University of Malaysia Sarawak, Sarawak, Malaysia

**Keywords:** adolescent, mental health, psychological distress, adaptation

## Abstract

**Background:**

Academic stress is part of a student’s life. Chronic stress may result in mental health problems, affecting the adolescent’s well-being in adulthood. However, not all types of stress result in a negative effect. Therefore, understanding how adolescents adapt to academic stress can lay the groundwork for preventive interventions. The Response to Stress Questionnaire (RSQ) for academic problems centred on a multidimensional model of responses to stress. However, it has not been tested among Malaysians. Thus, this study aimed to validate the questionnaire among Malaysians.

**Methods:**

The questionnaire was translated into the Malay language using forward and backward translation. Data were collected via self-administered questionnaires at a secondary school in Kuching. A validity test was conducted using face and content validation by subject matter experts, and construct validation was performed using exploratory factor analysis (EFA). A reliability test was conducted by checking Cronbach’s alpha.

**Results:**

Results showed that the questionnaire has good validity and reliability. The EFA resulted in only three dimensions of responses to stress among Malaysian adolescents in contrast to the five dimensions in the original RSQ for academic problems. The Cronbach’s alpha showed good reliability of the questionnaire.

**Conclusion:**

The questionnaire measuring responses to stress was valid and reliable in assessing the responses of adolescents to academic stress.

## Introduction

Academic-related stress remains a critical problem for all students. The education system has loaded students with various pressures, such as extensive curriculum, examination anxiety, neck-to-neck competitions and expectations and pressure from parents and teachers that add tons to their problems. Adolescents are the budding future of a nation. Studies about adolescents’ experiences and how they respond to, ponder about and handle stressful events (academic stress) can lay the groundwork for preventive interventions. These interventions are designed to help adolescents avoid stressful conditions, modify their way of appraising stress, find useful social resources or improve their adaptive coping capability (1, 2). The impact of ongoing stress, particularly academic-related stress, on adolescents’ well-being remains underexplored. Understanding why students use a certain coping strategy is crucial to health education, especially at the stage of adolescence when they are developing habits that can influence their future lifestyle choices (3).

Different researchers characterised coping differently. Certain researchers viewed coping as goal-directed efforts (problem-focused coping) aimed either at resolving the stressor and its environment or alleviating the negative feelings that emerge as a consequence of stress (emotion-focused coping) (4). Another perspective viewed primary and secondary voluntary coping strategies as directed at preserving, enhancing or modifying control over the situation and oneself to alter stressful events and adapting to existing circumstances, respectively, and referred to relinquished control as the absence of any coping effort (5). Skinner and Wellborn’s motivational model of psychological control and coping centred on simple human desires for competency, autonomy and relatedness (6). According to Skinner (7), coping includes voluntary and involuntary reactions to cope with threats to competency, autonomy and relatedness. In contrast, Eisenberg et al. characterised coping as a subgroup of the larger group of self-control (8). They distinguished coping into three self-control aspects: i) efforts to control the condition (e.g. problem-focused coping); ii) efforts to control feelings directly (e.g. emotion-focused coping) and iii) efforts to control emotionally driven behaviour (e.g. behaviour control) (8). Compas et al. (9–11) viewed coping as one facet of a larger set of processes performed when dealing with stress. They suggested that stress reactions can be observed alongside two distinct dimensions: voluntary against involuntary and engagement against disengagement.

Readily available questionnaires on coping with stress were created for grown-ups, children and teenagers with minimal or no adjustment. No agreement has been reached regarding the stress coping dimensions that best distinguish various coping skills used by children and adolescents (12). The most common coping dimensions described were problem-focused as opposed to emotion-focused coping, primary against secondary coping and engagement set against disengagement coping. These dimensions had been applied in various studies on child and adolescent coping, adding to the confusion and difficulty in incorporating findings across studies (12). Nonetheless, a review on coping instruments used in numerous studies had concluded that coping in childhood and adolescence is multidimensional (12).

The Response to Stress Questionnaire (RSQ) centres on the multidimensional model of responses to stress and highlights the significance of evaluating various reactions to stress, involving voluntary (controlled coping response) and involuntary responses (automatic response) (12). The primary component in this model distinguishes between voluntary and involuntary responses to stress. Voluntary coping refers to efforts made within one’s deliberate consciousness and is targeted at managing one’s thinking, behaviour, emotion or physiological reactions to stressful events. In contrast, involuntary coping refers to responses that may or may not be within one’s conscious awareness, for example intrusive thoughts, rumination, emotional numbing and emotional and physiological arousal (12). Then, voluntary and involuntary responses to stress are further divided into subsequent aspects of engagement with or disengagement from the source of stress (12). Engagement coping refers to reactions targeted at the stressor, whereas disengagement coping aims at avoiding the stressor. As voluntary stress coping strategies are goal-oriented, they can be divided further into primary (directed at changing the stressor or its situation or one’s emotional response to the source of stress) and secondary voluntary coping strategies (aimed at adapting to the stressor). However, only engagement coping strategies were found to differ in terms of primary versus secondary voluntary responses. This finding could be due to the difficulty in asserting primary control through disengagement coping responses (12).

The RSQ was created to develop and evaluate children’s and adolescents’ responses to specific stress, such as academic stress, social stress and family conflict. To expedite the recollection of memory and increase the reliability, the RSQ can assist respondents to relate to specific stressful occurrences which are the aim of coping attempts (12). Thus, this research used the RSQ for academic problems research to examine the coping mechanisms of adolescents for academic stress. However, a validated RSQ for academic problems is currently not available in the Malay language. Thus, this research was conducted to validate the RSQ for academic problems among adolescents attending secondary school in Kuching Division.

## Methods

### Samples

This study used a cross-sectional research method involving adolescents attending secondary school in Kuching Division. A secondary school from Kuching District Education Office was purposively selected for this study. The questionnaire was distributed to 200 participants for factor analysis and internal consistency reliability (Cronbach’s alpha) test. The sample size was sufficient for factor analysis (13, 14).

### Procedure

The research was endorsed by the Research Ethics Committee of the Universiti Malaysia Sarawak (UNIMAS) and the Ministry of Education Malaysia. Eight classes from the school were randomly selected and 25 students from each class were also randomly selected to participate in the study. All participants were briefed about this research and required to sign a parental consent form before they answered the questionnaire. The inclusion criteria were Malaysian citizen, aged 13 years old–19 years old and currently studying in a government-funded secondary school in Kuching Division. Students who were diagnosed with mental illness were excluded from the study. Self-administered questionnaires were distributed through the class teacher.

### Research Instrument

The questionnaire developed consists of three parts: i) the respondent’s socio-demographic characteristics; ii) the psychological distress of the respondent using Kessler’s Psychological Distress Scale (K10) questionnaires and iii) the respondent’s coping mechanism with academic stress. K10 is a popular tool for mental illness assessment in clinical and community settings (15, 16). The Malay version of K10 had been developed and validated among the Malaysian population (17).

The original RSQ for academic problems was translated into Malay using forward and backward translation. The procedure of translation and cross-cultural adaptation of the questionnaire was performed according to the recommendations (18–20). The first part of translating the English questionnaire into Malay was done separately by two subject experts whose mother tongue was Malay and who were also well-versed in English. Then, the two forward translations were compared and merged by an independent translator. Back translation into English was done independently by another two subject experts who were well-versed in English and Malay. The back translators did not have a priori understanding of the intent of the original instrument. The final Malay version of the RSQ was reviewed by a committee consisting of the researcher and a clinical psychologist. The committee compared the back translations with the original text, identified discrepancies and discussed with the translators whether any changes were needed. The committee also evaluated the introduction and instruction of the questionnaire and reviewed the scale of answers for each question before deciding on the final Malay version of the questionnaire (19).

The first part of this questionnaire asked for a list of academic-related issues that the respondents find stressful to deal with during the past 6 months. This part is set to help respondents think of specific examples of academic stress before the survey continues to assess how they coped with stress in the second part. Ten items were measured using a 4-point Likert scale (1–4) ranging from ‘not at all’ to ‘very’. The scores were summed up, and the higher the score, the higher the academic stress level experienced by the respondent. The second part of the questionnaire measured the coping approaches used by the respondents in response to academic stress. The responses to stress encountered by the respondents during the past 6 months were assessed using 57 items. Theoretically, the RSQ consists of 19 three-item subscales grouped into five hypothesised factors: i) primary voluntary; ii) secondary voluntary; iii) disengagement; iv) involuntary engagement and v) involuntary disengagement coping strategies. The Cronbach’s alpha for each factor ranged from 0.73 to 0.89 (12). Each of the 57 items is measured in 4-Likert scale from 1 to 4 (1 = not at all to 4 = very). Higher scores on factors indicates a greater inclination of the adolescents to use a strategy or strategies in response to stress.

### Face and Content Validation

Five subject matter experts who were psychiatrists and clinical psychologists were consulted to validate the content of the questionnaire. The consultation was performed using a non-face-to-face approach, which was more efficient than the face-to-face approach in terms of cost, time and response rate (21). The panel reviewed the questionnaire to ensure its suitability for the intended purpose. Corrections were made to simplify the questions. Then, a standardised validation form was developed and emailed to the experts together with the explicit instructions and objective of the questionnaire to ensure the apprehension of content validation. A rating scale of relevance (1–4), ranging from non-relevant to highly relevant, was employed to rate the degree of relevance for each item (21). The experts were required to review the questionnaire objectively and encouraged to state their comments to improve the relevance of the items (21, 22). A content validity index (CVI) was employed to evaluate the content validity of the questionnaire. The CVI was calculated using the item-level CVI (I-CVI) and scale-level CVI, namely the average S-CVI (S-CVI/Ave) and universal agreement S-CVI (S-CVI/UA) (23, 24).

### Data Analysis

Microsoft Excel 2010 was used to compute I-CVI and S-CVI/Ave and S-CVI/UA. The collected data were coded and analysed for exploratory factor analysis (EFA) and internal reliability using IBM SPSS version 23.0. EFA is a commonly used analysis to explore theoretical constructs, define a questionnaire’s factorial structure and explore items that constitute a particular construct collectively (25). The internal reliability using Cronbach’s alpha was used to determine whether the questionnaire adopted provided reliable readings (26).

### Ethical Consideration

Ethical approval for this study was obtained from the Research Ethics Committee of UNIMAS. Parental consent was obtained from participants below 18 years old before conducting the research, whereas those aged 18 years and above were given informed consent detailing the purpose and benefits of the study, absolute privacy of data obtained and the right to withdraw from the research at any time without affecting their current situation.

## Result

### Content Validation

The computation showed that I-CVI ranged from 0.80 to 1.00, indicating that the items were relevant (23). The S-CVI/Ave and S-CVI/UA were above 0.90 and 0.80, respectively, showing good content validity (27) (Table 1).

### Initial Principal Component Analysis

The RSQ for academic problems had 57 items coded as D1 to D57. The mean score for each item ranged between 1.55 and 2.54, whereas the standard deviation of the scores ranged between 0.794 and 1.056.

The initial principal component analysis (PCA) was run to check the appropriateness of conducting PCA on the correlation matrix. A sample size of 195 was deemed adequate as the minimum sample size for factor analysis should be 50 (14). The correlation matrix table showed that the correlations between variables were more than 0.30, indicating that the factor analysis was appropriate. The Kaiser-Meyer-Olkin Measure of Sampling Adequacy (KMO MSA) was 0.923, indicating excellent sampling adequacy for factor analysis (28). Bartlett’s test was significant with χ^2^ (1596) = 7315.430, *P*-value < 0.001. This result showed that the sample correlation matrix differed significantly from an identity matrix (29).

**Figure 1:**
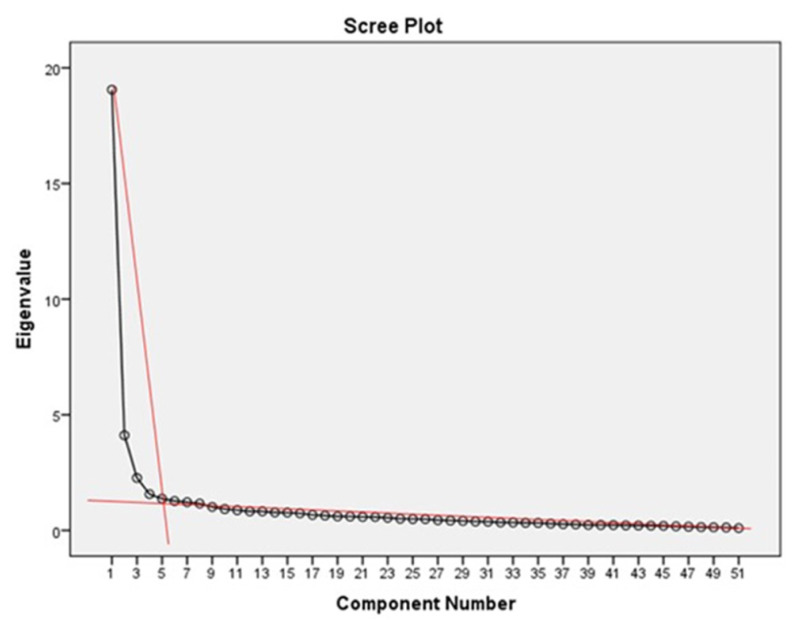
The Scree Plot for RSQ construct extracted three components

Several methods were applied to determine the number of factors to extract from the EFA. First, the latent root criterion was used and found that 11 components had Eigenvalues greater than 1 (14). Second, a scree plot (Figure 1) was used to visualise that three to four components emerged for this construct (14). Third, parallel analysis (30) was used where a set of random eigenvalues was simulated using an online parallel analysis engine (31). The eigenvalues from the data were compared against the random eigenvalues where data with eigenvalues greater than the randomly generated eigenvalues will be retained. Using the parallel analysis, three components were retained.

EFA was run using PCA with extraction to fixed factors of three and four, a varimax rotation and suppressing coefficients less than 0.40 (14). After comparing the analysis results for three and four components, the three-component model was a better fit.

### Exploratory Factor Analysis for Response to Stress Questionnaire for Academic Problems

The EFA was conducted using the PCA extraction method with varimax rotation on the 57 items measuring the RSQ. The result of KMO MSA was 0.923 and Bartlett’s Test of Sphericity was significant with χ^2^ (1296) = 7315.43, *P*-value < 0.001. These results indicate that the data were suitable to continue with the data reduction procedure (14).

The factor loadings for items D2, D27, D41 and D56 were less than 0.45 (14) and thus were removed from the analysis, whereas two items with cross-loadings of more than 75% (D23 and D37) were also removed. Fifty-one items were retained in the final model with three components explaining the total variance of 49.88 (Table 2). Table 3 presents the final EFA model.

### Reliability Analysis of the Questionnaire

The K10, academic stress scale and responses to stress scale from the RSQ were tested for internal consistency of the scales among the Malaysian secondary school students. These tools had good to excellent values of internal consistency (Table 4). Finally, the internal consistency of the respective components measuring RSQ was analysed (Table 5). The items in all three components have good internal consistencies.

## Discussion

This research aimed to validate the RSQ among adolescents attending secondary school in Kuching Division. The data used in this study were suitable for conducting a valid EFA procedure using the outputs from the descriptive statistics analysis. The sample size of 195 was satisfactory for EFA as the researchers stated that the minimum sample size for factor analysis should be 50 (14). From the EFA results for the responses to the stress construct of RSQ, the data met Bartlett’s test (significant); the KMO is more than 0.6 and the factor loadings exceeded the minimum threshold of 0.4 (14). The measuring instrument explained a total of 49.881% in the structure of interactions among the items. This result was slightly below the acceptable level as Streiner (32) stated that the total variance explained by the retained components should be at least 50%. Hair et al. (14) also mentioned that in social sciences, a 60% of total variance or less can be considered as satisfactory. These results revealed that the elements were applicable in this study. The results of this study also showed that the questionnaire was stable and internal consistency was acceptable (Cronbach’s alpha > 0.7) for each part of the questionnaire and the dimensions in the responses to stress scale of the RSQ.

The EFA results showed only three dimensions of responses to academic stress among the adolescents, compared with the five dimensions proposed in the original RSQ. Connor-Smith and his colleagues (12) conceptualised that coping with stress involved voluntary and involuntary responses. Voluntary coping would be distinguished along a dimension of engagement versus disengagement, and voluntary engagement coping would be further differentiated into primary and secondary voluntary coping. Involuntary coping would be distinguished into engagement and disengagement coping (12). However, in this research, involuntary coping failed to distinguish between engagement and disengagement coping. The result of this study identified engagement and disengagement coping within the voluntary coping dimension. Further division of voluntary engagement coping into primary and secondary coping was not seen in this study.

In this study, the items identified in component 1 (involuntary coping) belonged to involuntary engagement and disengagement coping of the original dimensions, except items 34 and 52 (items in secondary voluntary engagement coping). This finding indicates that the EFA result failed to distinguish between engagement and disengagement coping, and two items were misclassified into component 1. The items in component 2 (voluntary engagement coping) were from primary and secondary voluntary engagement coping of the original RSQ. In this study, no distinctions were noted between primary and secondary voluntary engagement coping. For component 3 (voluntary disengagement coping), the five items from the original dimension were loaded into the same component in this study with six misclassified items.

The failure of the EFA to replicate the conceptualised dimension could be due to the difference in the culture of this research setting as the RSQ has not been used in East Malaysia. East Malaysian adolescents, particularly Sarawakian students, are likely to experience different types and rates of recurrence of stressors, the apparent stressfulness of the negative events, the tolerability of different reactions to stress and the availability of coping resources due to their unique cultural differences. Variations in factor constructs attained across samples and cultures may be due to the highly contextual nature of coping, which relies on the interaction between people, the source of stress and the environments (33).

The problems linked to disagreement between factors and/or misclassification could be due to the sample size (34). It was suggested that by increasing the sample size, the sampling error could be reduced, and factor analysis solutions could be more stable and reliable (35). Moreover, the RSQ has been validated using confirmatory factor analysis in various settings and cultures: Spanish college students (36), Navajo adolescents (37) and Chinese university students (36).

A limitation of this research was that it was conducted in only one secondary school in Kuching Division and utilised data from only 195 randomly selected adolescents. This shortcoming can be addressed by future studies by considering additional samples from public and private schools. Thus, whether the validated measures of response to stress (RSQ) in this study will be the same in other adolescents attending secondary school can be determined.

## Conclusion

From the EFA results, this research has accomplished a modest milestone with a three-dimension structure of measuring responses to stress. Six items were deleted from the original 57 items because their factor loadings were lower than the defined threshold in this study (0.45) and the cross-loadings. The three dimensions of responses to stress were involuntary, voluntary engagement and voluntary disengagement coping strategies. The questionnaire has shown to be an appropriate tool designed for its purpose.

## Figures and Tables

**Table 1:** Content Validity Index (CVI) for the questionnaire (*n* = 195)

Measure	Number of items	I-CVI	S-CVI/Ave	S-CVI/UA
RSQ	57	0.80–1.00	0.97	0.84

**Table 2:** The number of components and total variance explained for RSQ (*n* = 195)

Component	Initial Eigenvalues			Rotation sums of squared loadings		
	
	Total	Variance (%)	Cumulative (%)	Total	Variance (%)	Cumulative (%)
1	19.057	37.367	37.367	10.549	20.683	20.683
2	4.114	8.067	45.434	8.960	17.568	38.251
3	2.268	4.447	49.881	5.931	11.630	49.881

**Table 3:** Varimax-rotated component analysis factor matrices (final model) (*n* = 195)

	Component		

	1	2	3
D57	0.789		
D47	0.735		
D49	0.718		
D40	0.690		
D35	0.688		
D55	0.682		
D53	0.671		
D33	0.668		
D46	0.644		
D34	0.638		
D51	0.624		
D44	0.621		
D31	0.608		
D22	0.605		
D25	0.577		
D26	0.575		
D38	0.560		
D28	0.523		
D18	0.508		
D6	0.500		
D12	0.493		
D42	0.482		
D52	0.468		
D30		0.792	
D50		0.754	
D39		0.746	
D19		0.717	
D21		0.679	
D45		0.661	
D17		0.652	
D32		0.652	
D7		0.623	
D24		0.592	
D43		0.592	
D20		0.579	
D3		0.579	
D54		0.545	
D36		0.541	
D48		0.533	
D29		0.521	
D1			0.680
D10			0.650
D15			0.611
D4			0.606
D13			0.581
D11			0.551
D16			0.544
D14			0.534
D9			0.532
D8			0.505
D5			0.475

Notes: Extraction method = PCA; Rotation method = Varimax with Kaiser Normalisation; Rotation converged in seven iterations

**Table 4:** Internal consistencies of scales for Kessler’s Psychological Distress Scale and RSQ (*n* = 195)

Instrument	Number of items	Cronbach’s alpha
Kessler’s Psychological Distress Scale (K10)	10	0.912
RSQ Academic stress	10	0.839
RSQ Response to stress	51	0.965

**Table 5:** The reliability analysis for every component measuring RSQ construct (*n* = 195)

Component	Name of component	No. of items	Cronbach’s alpha
1	Involuntary coping	23	0.948
2	Voluntary engagement coping	17	0.933
3	Voluntary disengagement coping	11	0.885
